# Relationship between serum leptin level and laboratory and anthropometric indices of malnutrition in patients on hemodialysis

**DOI:** 10.4103/0971-4065.43689

**Published:** 2008-07

**Authors:** F. Ahamadi, R. Bosorgmehr, E. Razeghi

**Affiliations:** Department of Nephrology, Tehran University of Medical Sciences, Tehran, Iran

**Keywords:** Anthropometric parameters, hemodialysis, leptin, malnutrition

## Abstract

Protein-energy malnutrition is a major problem and one of the risk factors for mortality in hemodialysis patients. There is no single index in evaluation of nutritional status in these patients, so leptin can be used as one of the parameters. In this study, the correlation between serum leptin with biochemical and anthropometric parameters of nutrition has been evaluated. This cross-sectional study has been performed on 60 hemodialysis patients (32 males and 28 females) in 2006. The patients on hemodialysis for under 1 year and who has a history of consumption of lipid lowering agents or glucocorticoids, or an infectious or inflammatory disease were excluded. Malnutrition laboratory parameters and serum leptin were measured before hemodialysis. Serum leptin was measured with enzyme-linked immunosorbent assay (ELISA) method with direct dbc kit and malnutrition laboratory parameters measured with standard laboratory methods, patients anthropometric parameters evaluated after hemodialysis. The mean age of patients was 47.5 ± 16.1 years and the range of serum leptin level was 0.6-64.8 ng/ml. Mean serum leptin level were 22.64 ± 19.54 ng/ml in females and 16.74 ± 20.16 ng/ml in males on hemodialysis and in spite of higher level of leptin in females there was not any statistically significant difference between females and males serum leptin. Absolute value of correlation coefficient between serum leptin and anthropometric parameters and most laboratory parameters was < 0.25 (except ferritin, iron, phosphorous in males and total protein, hemoglobin, urea, and creatinin in females which was between 0.25 and 0.50). Our results suggest that the increased serum leptin level does not have a major role in diagnosis of malnutrition in hemodialysis patients and there is a poor correlation between malnutrition parameters and serum leptin level.

## Introduction

Protein-energy malnutrition is a common problem in hemodialysis patients, which increases their morbidity and mortality and decreases life expectancy as a result of accompanying infection and cardiovascular diseases.^1^

Generally, there are multiple causes contributing to malnutrition in hemodialysis patients. These include restricted diet, metabolic acidosis, gastroparesis, appetite suppression as a side effect of the drugs, chronic volume overload, presence of an acute or chronic systemic diseases which cause inflammatory responses and dialysis itself. Therefore, nephrologists suggest the initiation of hemodialysis before malnutrition has set in.^2^

There is no single index to determine malnutrition in dialysis patients and malnutrition evaluation is done by multiple parameters which need lots of money and time.

According to the studies, 40-70% of end-stage renal-disease (ESRD) patients are malnourished which is due to multiple factors. One of the causes of malnutrition is anorexia which may have different reasons.^3^

Leptin is a 16-kDa protein hormone and a product of obesity gene (ob) which is produced exclusively by adipocytes and its main effect on hypothalamus that decreases appetite, increases energy expenditure, and reduces weight.^4^,^5^ According to the studies, it seems that increase of serum leptin in patients on chronic hemodialysis may cause anorexia and malnutrition.^6^

The exact reason of serum leptin level elevation in ESRD is not known and factors like decrease in renal clearance and erythropoietin level and chronic inflammation, have been thought to be the contributing factors.^5^ Some studies showed a positive correlation between serum leptin and some malnutrition parameters.^7^–^10^ But this correlation was not seen in one of them^11^ and on the contrary, leptin was introduced as a marker of good nutritional status in hemodialysis patients in another study.^12^ Due to the different results of studies, the definite role of leptin as a marker of malnutrition in hemodialysis patient is not definite. This study was designed and performed to investigate the correlation between serum leptin level and malnutrition parameters in hemodialysis patients.

## Materials and Methods

Our study was a cross-sectional study done on hemodialysis patients in Emam Khomeini Hospital and Sina Hospital in Tehran in 2006.

The sample size of the study was calculated by comparison of mean serum leptin in patients with and without malnutrition defined by one of the markers of malnutrition in hemodialysis patients backed up by the study of Koo *et al.*^12^ (including 60 hemodialysis patients). Patients entered the study by ordinal random sampling if they meet the following criteria: patient's age must be between 15 and 80 years, patients must be on hemodialysis at least twice a week and 4 h each time. Patients who were on hemodialysis for < 1 year and the ones who were taking lipid lowering agents, glucocorticoids, who had active infection or inflammatory diseases and patients who had been hospitalized during the last 3 months before entering the study were excluded.

An informed consent was obtained from all subjects. We explained the importance of malnutrition to patients in their life quality before the examinations were done. A blood sample was taken from each patients before hemodialysis and hemoglobin, quantitative C-reactive protein (CRP), calcium, phosphorous, parathyroid hormone, urea, creatinine, serum iron, total iron binding capacity, ferritin, transferrin, total protein, albumin, triglyceride, cholesterol, low-density lipoprotein (LDL), high-density lipoprotein, and serum leptin were measured. Serum leptin was measured using enzyme-linked immunosorbent assay method by dbc Direct Kit (Diagnostic Biochem, Canada) with 0.5 ng/ml sensitivity. Intraassay coefficient of variation (CV) and interassay CV of the kit were 7.4 and 8.7%, respectively. After the measurement of leptin, adjusted leptin was calculated by dividing serum leptin by body mass index (BMI). The other laboratory tests were done through standard laboratory methods.

The following anthropometric parameters were measured 10-20 min after hemodialysis was fulfilled.

*Usual body weight (UBW)*: mean body weight of patients during last 6 months using the data of their clinical files.

*Height*: measuring from the vertex to the heel (using tape measure)

*Body mass index (BMI)*: The patients body weight in kilograms divided by their square height in meter.

*Triceps skin fold (TSF) and biceps skin fold (BSF)*: skin-fold thickness amount in triceps and biceps region (by adipometer skin-fold caliper).

*Mid arm circumference (MAC)*: arm circumference measured in midarm between acromyon and olecranon process (using tape measure).

*Mid arm muscle circumference (MAMC)*: Was calculated as follows:

MAMC = MAC − (3.14 × TSF)

*KT*/*V* index which is an indicator for adequacy of dialysis was measured by multiplying clearance of urea by time divided by volume of distribution of urea.

The hypothetical cut-offs for defining malnutrition are shown in Table 1.

**Table 1:** Hypothetical cut point for definition of malnutrition for some biochemical and anthropometric parameters

Parameter	Cut point	Malnutrition limit
BMI (kg/m2)	18.5	<18.5
TSF (mm)		
Male	6	<6
Female	8	<8
MAC (cm)		
Male	26	<26
Female	24	<24
MAMC (cm)		
Male	20	<20
Female	18	<18
Albumin (g/dl)	4	<4
Ferritin (ng/ml)	100	>100
Transferrin (mg/dl)	200	<200
Hemoglobin (g/dl)	10	<10
Cholesterol (mg/dl)	150	<150
Triglyceride (mg/dl)	150	<150
CRP (quantitative) (mg/l)	10	>10
ESR (mm/h)	20	>20
Calcium (mg/dl)	8	<8
Phosphorous (mg/dl)	3	<3
PTH (pg/ml)	100	>100
Creatinine (mg/dl)	8	<8
*KT/V*	1.2	<1.2

BMI: Body mass index, TSF: Triceps skin fold, MAC: Mid arm circumference, MAMC: Mid arm muscle circumference, CRP: C-reactive protein, ESR: Erythrocyte sedimentation rate, PTH: Parathyroid hormone

Statistical analysis was done on data gathered using SPSS-11.5 software. To evaluate the correlation of variables considering whether they are quantitative or qualitative and type of their correlation parametric statistical tests (analysis of variance, ANOVA, *T*-test, and Pearson test) and nonparametric statistical tests (Mann-Whitney and Spearman's rho test) were used.

## Results

A total of 60 patients including 32 males (53.3%) and 28 females (46.7%) with mean age of 47.5 ± 16.1 years were investigated. Mean amount of measured parameters for each gender are presented in Table 2.

**Table 2:** Mean value of laboratory and anthropometric parameters in hemodialysis patients and gender differences of them

Variables	Mean (*N* = 60)	Gender difference		Significance* (*P*-value)
		
		Male (*n* = 32)	Female (*n* = 28)	
Leptin (ng/ml)	19.49 ± 19.93	16.74 ± 20.16	22.64 ± 19.54	0.256
Adjusted leptin	0.86 ± 0.94	0.73 ± 0.88	1.03 ± 1	0.23
Age (years)	47.45 ± 16.08	44.72 ± 16.97	50.57 ± 14.67	0.16
Duration of hemodialysis (months)	66.90 ± 53.36	69.81 ± 59.55	63.57 ± 46.15	0.65
Height (m)	161.37 ± 13.10	167.19 ± 14.10	154.71 ± 7.78	0.000
UBW (kg)	65.05 ± 12.17	66.32 ± 21.41	63.58 ± 11.94	0.389
BMI (kg/m2)	23.47 ± 4.53	22.56 ± 2.57	24.51 ± 5.94	0.098
TSF (mm)	12.13 ± 4.42	11.16 ± 2.79	13.25 ± 5.59	0.067
BSF (mm	6.35 ± 3.58	4.97 ± 1.65	7.93 ± 4.48	0.001
MAC (cm)	26.32 ± 3.91	25.88 ± 3.35	26.82 ± 4.47	0.354
MAMC (cm)	22.49 ± 3.15	22.43 ± 2.96	22.56 ± 3.41	0.871
Serum albumin (g/l)	4.42 ± 0.39	4.56 ± 0.36	4.26 ± 0.35	0.002
Total protein (g/l)†	7.30 ± 0.60	7.13 ± 0.48	7.50 ± 0.68	0.034
Serum iron (µg/dl)	125.58 ± 51.87	124.94 ± 54.78	126.32 ± 49.32	0.919
Ferritin (ng/ml)	707.75 ± 658.08	631.05 ± 543.99	795.40 ± 769.18	0.339
TIBC (µg/dl)	308.12 ± 46.63	311.28 ± 51.81	304.50 ± 40.54	0.579
Transferrin (mg/dl)	1265.42 ± 1235.60	1427.97 ± 1219.07	1079.64 ± 1250.05	0.280
*T* sat. ratio (%)	42.93 ± 21.28	42.39 ± 22.14	43.55 ± 20.64	0.836
Total cholesterol (mg/dl)	149.57 ± 37.08	135.59 ± 29.14	165.54 ± 39.16	0.001
LDL (mg/dl)	86.43 ± 26.16	77 ± 18.79	97.21 ± 29.39	0.002
HDL (mg/dl)	30.58 ± 9.13	28.88 ± 8.25	32.54 ± 9.82	0.122
Triglyceride (mg/dl)	164.78 ± 72.33	146.5 ± 62.98	185.68 ± 77.64	0.035
Urea (mg/dl)	75.10 ± 48.31	74.31 ± 45.58	76 ± 52.08	0.894
Creatinine (mg/dl)	5.52 ± 3.43	5.76 ± 3.42	5.24 ± 3.49	0.561
Hemoglobin (g/dl)	9.60 ± 2.07	9.55 ± 2.53	9.66 ± 1.41	0.839
ESR (mm/h)	63.42 ± 46.66	53.06 ± 36.6	74.89 ± 54.11	0.072
CRP (mg/l)	12.60 ± 17.17	10.89 ± 12.19	14.55 ± 21.06	0.415
Phosphorous (mg/dl)	6.60 ± 5.14	5.99 ± 1.5	7.29 ± 7.36	0.332
PTH (pg/ml)	198.82 ± 363.70	260.34 ± 410.94	128.51 ± 292.5	0.163
Calcium (mg/dl)	8.39 ± 0.69	8.45 ± 0.83	8.33 ± 50	0.507
*KT*/*V*	1.25 ± 0.23	1.21 ± 0.22	1.3 ± 0.24	0.154

Results are shown as mean ± SEM

* Student's *t*-test.

† *T*-sat ratio: Transferrin saturation ratio

UBW: Usual body weight, BMI:- Body mass index, TSF: Triceps skin fold, BSF: Biceps skin fold, MAC: Mid arm circumference, MAMC: Mid arm muscle circumference, LDL: Low-density lipoprotein, CRP: C-reactive protein, PTH: Parathyroid hormone, ESR: Erythrocyte sedimentation rate, TIBC: Total iron binding capacity, HDL: High-density lipoprotein

**Fig. 1:**
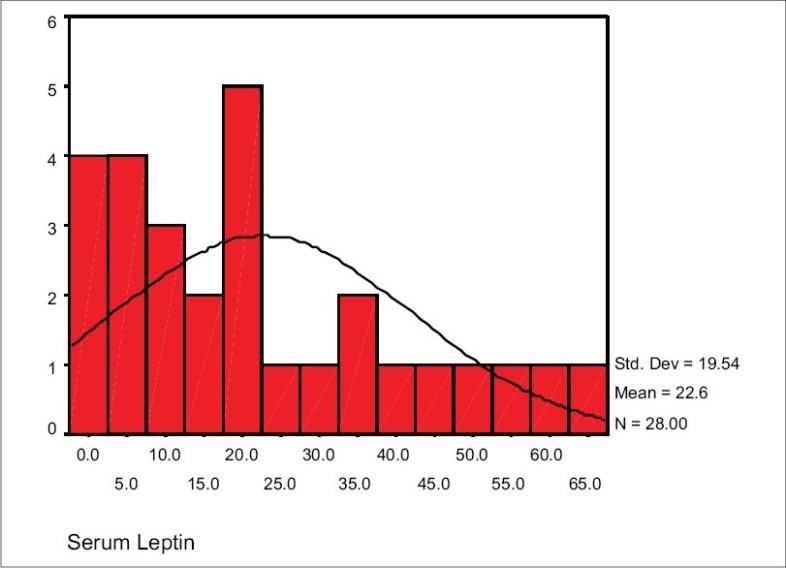
Normal distribution curve for serum leptin level in hemodialysis women

**Fig. 2:**
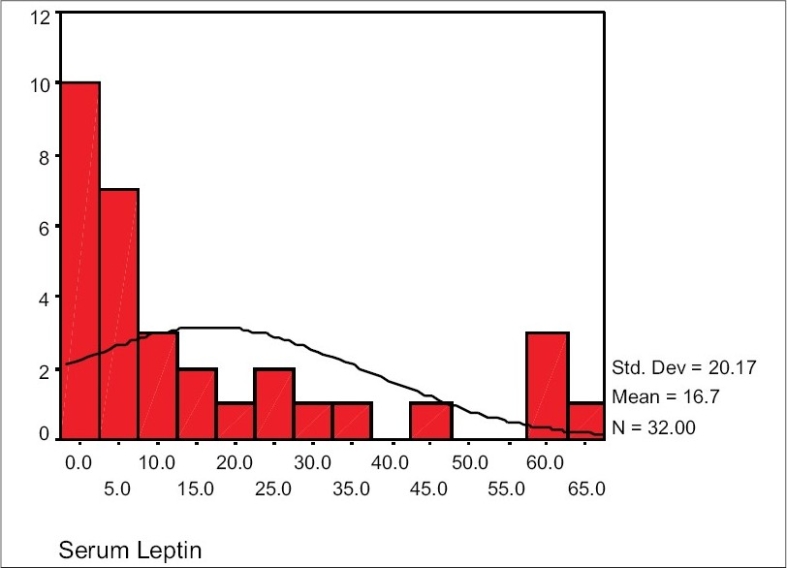
Normal distribution curve for serum leptin level in hemodialysis men

The range of serum leptin of hemodialysis patients in our study was 0.6-64.8 ng/ml. Mean serum leptin in women and men were 22.64 ± 19.54 ng/ml and 16.74 ± 20.16 ng/ml respectively and their difference was not statistically significant [Figs. 1 and 2].

Pearson correlation coefficient for serum leptin level and adjusted leptin with malnutrition laboratory and anthropometric parameters and age, and hemodialysis duration are shown for both genders [Table 3].

**Table 3:** Pearson correlation coefficient for serum leptin level and adjusted leptin with malnutrition parameters in hemodialysis patients

Adjusted leptin	Serum leptin		Variables	
		
	Male	Female	Male	Female
Age (years)	0.13	0.22	0.12	0.22
Duration of hemodialysis (months)	0.03	0.28	0.06	0.31
Height (m)	0.28	−0.10	0.26	−0.15
UBW (kg)	0.28	−0.25	0.24	−0.42*
BMI (kg/m2)	0.16	−0.23	0.09	−0.43*
TSF (mm)	−0.08	−0.09	−0.11	−0.27
BSF (mm)	0.08	0.08	0.04	−0.13
MAC (cm)	0.14	−0.06	0.08	−0.27
MAMC (cm)	0.23	−0.004	0.16	−0.19
Serum albumin (g/l)	0.15	−0.09	0.13	−0.11
Total protein (g/l)	−0.06	0.30	−0.07	0.35
Serum iron (µg/dl)	0.33	−0.25	0.35*	−0.11
Ferritin (ng/ml)	0.38*	−0.10	0.41*	−0.03
TIBC (µg/dl)	−0.21	0.16	−0.21	0.03
Transferrin (mg/dl)	−0.06	−0.07†	−0.04	−0.13†
*T* sat. ratio (%)	0.33	−0.22	0.35*	−0.08
Total cholesterol (mg/dl)	−0.22	0.01	−0.22	−0.03
LDL (mg/dl)	−0.17	−0.04	−0.17	−0.07
HDL (mg/dl)	−0.09	0.13	−0.09	0.13
Triglyceride (mg/dl)	−0.11	−0.04	−0.12	−0.21
Urea (mg/dl)	−0.19	0.37*,†	−0.19	0.12†
Creatinine (mg/dl)	−0.15	0.35*,†	−0.16	0.22†
Hemoglobin (g/dl)	−0.04	−0.31	−0.04	−0.31
ESR (mm/h)	0.12	0.11	0.13	0.14
CRP (mg/l)	−0.03	−0.05†	−0.02	−0.10†
Phosphate (mg/dl)	−0.28	−0.02†	−0.26	−0.12†
Calcium (mg/dl)	0.25	0.12	0.26	−0.001
PTH (Pg/ml)	−0.23	−0.18†	−0.20	−0.18†
*KT/V*	−0.07	0.28	−0.05	0.16

* *P* < 0.05

† Spearman's rho test

UBW: Usual body weight, BMI: Body mass index, TSF: Triceps skin fold, BSF: Biceps skin fold, MAC: Mid arm circumference, MAMC: Mid arm muscle circumference, LDL: Low-density lipoprotein, CRP: C-reactive protein, PTH: Parathyroid hormone, ESR: Erythrocyte sedimentation rate, TIBC: Total iron binding capacity, HDL: High-density lipoprotein

Comparison of mean serum leptin level and some malnutrition parameters considering the hypothetical cut points are shown in Table 4. After dividing the patients to diabetic and nondiabetic subgroups and evaluation of correlation between leptin and other malnutrition parameters in each subgroup, the correlation was detected only in nondiabetic hemodialysis patients between age and serum leptin (*r* = 0.38, *P* = 0.007) and correlation with other variables were not significant. The correlation between leptin and other variables was not significant in diabetic subgroup.

**Table 4:** Mean serum leptin in malnutrition parameters considering the defined cut point in each gender in HD

Parameter	Gender	Cut point	Number of patients	Mean serum leptin	SD	*P* value
BMI (kg/m^2^)	Male	<18.5	5	16.18	25.121	0.95
		≥18.5	27	16.85	19.69	−
	Female	<18.5	9	23.54	19.73	0.87
		≥18.5	19	22.22	19.98	−
TSF (mm)	Male	<6	0	−16.74	−20.17
		≥6	32	−	−	−
	Female	<8	4	26.32	22.17	0.73
		≥8	24	22.03	19.53	
MAC (cm)	Male	<26	14	14.54	20.28	0.59
		≥26	18	18.45	20.49	
	Female	<24	7	21.47	18.02	0.85
		≥24	21	23.04	20.43	
MAMC (cm)	Male	<20	7	11.65	20.68	0.47
		≥20	25	18.17	20.21	
	Female	<18	3	24.00	26.73	0.91
		≥18	25	22.24	19.22	
Albumin (g/dl)	Male	<4	2	6.7	6.08	0.15
		≥4	30	17.41	20.64	
	Female	<4	5	26.62	19.44	0.63
		≥4	23	21.78	19.89	
Ferritin (ng/ml)	Male	≤100	6	17.37	18.45	0.93
		>100	26	16.6	20.88	
	Female	≤100	4	24.5	26.01	0.88
		>100	24	22.34	18.95	
Transferrin (mg/dl)	Male	<200	6	14.167	23.00	0.73
		≥200	26	17.34	19.91	
	Female	<200	9	23.54	19.72	0.87
		≥200	19	22.22	19.98	
Hemoglobin (g/dl)	Male	<10	22	17.11	20.71	0.88
		≥10	10	15.94	19.98	
	Female	<10	11	30.08	23.19	0.14
		≥10	17	17.83	15.68	
Total cholesterol (mg/dl)	Male	<150	22	19.79	20.77	0.21
		≥150	10	10.04	17.93	
	Female	<10	8	25.30	24.01	0.7
		≥150	20	21.58	18.06	
Triglyceride (mg/dl)	Male	<10	19	15.411	21.66	0.65
		≥150	13	18.69	18.44	
	Female	<150	10	21.63	21.59	0.85
		≥150	18	23.21	18.96	
CRP (quantitative) (mg/l)	Male	>10	6	13.15	23.20	0.68
		≤10	26	17.57	19.82	
	Female	>10	9	18.87	15.72	0.44
		≤10	19	24.44	21.27	
ESR (mm/h)	Male	>20	22	20.55	22.57	0.11
		≤20	10	10.10	12.93	
	Female	>20	25	24.18	20.15	0.003
		≤20	3	9.86	2.63	
Calcium (mg/dl)	Male	<8	11	13.72	20.65	0.55
		≥8	21	18.33	20.24	
	Female	<8	8	20.15	17.47	0.034
		≥8	20	27.64	18.4	
Phosphorous (mg/dl)	Male	<3	1	30.7	−20.4	0.49
		≥3	31	16.3	−	−
	Female	<3	0	−22.64	−19.54	−
		≥3	28	−	−	−
PTH (pg/ml)	Male	≤100	17	20.55	22.98	0.25
		>100	15	12.43	16.11	
	Female	≤100	21	22.98	20.11	0.87
		>100	7	21.63	19.21	
Creatinine (mg/dl)	Male	<8	25	18.49	22.27	0.14
		≥8	7	10.48	7.72	
	Female	<8	20	17.53	16.24	0.025
		≥8	8	35.44	22.26	
KT/V	Male	<1.2	14	16.81	20.46	0.98
		≥1.2	18	16.69	20.53	
	Female	<1.2	11	10.4	10.62	0.016
		≥1.2	17	27.52	19.08	

SD: Standard deviation, BMI: Body mass index, TSF: Triceps skin fold, MAC: Mid-arm circumference, MAMC: Mid-arm muscle circumference, ESR: Erythrocyte sedimentation rate, CRP: C-reactive protein, PTH: Parathyroid hormone

## Discussion

According to the results of our study, mean anthropometric parameters in hemodialysis patients like UBW, BMI, BSF, TSF, MAC, and MAMC in women were more than in men. This difference was not significant for UBW, MAC, MAMC (*P* > 0.05); was marginally significant for BMI, TSF (*P* = 0.09, *P* = 0.06) and was significant for BSF (*P* = 0.001). Kayardi *et al.* came to the same results.^8^ It should be considered that the final conclusion about the upper limit of anthropometric parameters in women on hemodialysis can be reached only if we know the values of these parameters in general population and for each gender.

Our results showed that mean serum leptin and adjusted leptin in male and female patients were not significantly different (*P* > 0.05) (though mean serum leptin in hemodialysis women was more than men).

These results were different from the results of the study of Yilmaz *et al.* In their study, serum leptin level in women was significantly more than men.^9^

In our study, the correlation of BMI and UBW with serum leptin was negative and poor, the correlation of BMI and UBW with adjusted leptin in women was negative and moderate and in men was poor. In study of Kayardi *et al.*^8^ and Yilamz *et al.*,^9^ serum leptin and BMI had a good positive correlation. The different result of our study may be due to the lower ratio of patients having BMI < 18.5 in our sample group (23% patients). According to our results, there is a poor negative correlation between adjusted leptin and other anthropometric parameters. In previous studies^8^,^9^, there was a good positive correlation between these parameters. It seems that the reasoning of poor negative correlation of BMI and leptin can be extended for other anthropometric parameters too, as these parameters were within normal limits in most of our patients.

Our results demonstrate a poor positive correlation between serum leptin and patients age both in women and men differ and from the results of Yilamz *et al.*'s study.^9^ In their study, there was a moderate to good positive correlation between patients age (in both genders) and serum leptin. A significant positive correlation between patients age and serum leptin was seen in our study for nondiabetic subgroup (*P* = 0.007).

As in the study of Yilamz *et al.*,^9^ there was a poor correlation between dialysis duration and serum leptin in our study and it seems a correlation cannot be found between these two variables.

According to the results of our study, there is a poor correlation between leptin (and adjusted leptin) with hemoglobin, ESR, CRP, and albumin in patients of this study. The same findings are seen in study of Bossola *et al.*,^7^ Kayardi *et al.*,^8^ Koo *et al.*^12^ while Johansen *et al.*'s study^13^ showed a negative moderate correlation between albumin and leptin.

According to the results of our study, the mean of total cholesterol, LDL, and triglyceride in women is significantly more than those of men, but there was no correlation between leptin (and adjusted leptin) with these variables. Bossola *et al.*^7^ and Johansen *et al.*^13^ reached the same result, but in Svobodova study^10^ there was a significant positive correlation between serum leptin of hemodialysis patients and their cholesterol and triglycerides which is different from the results of this study.

Based on the results of our study, there is a positive moderate correlation between ferritin and iron with serum leptin (and adjusted leptin) in male patients. This correlation is not found in female patients. The correlation of ferritin and serum leptin may be due to the fact that ferritin is a acute phase protein and chronic inflammation is a probable cause of increased serum leptin level in ESRD patients.

All the patients of our study were clinically stable and did not have any acute diseases and were not taking corticosteroids. In Bossola *et al.*'s study,^7^ no significant correlation was detected between leptin and ferritin. In our study, there was no correlation between leptin and transferrin which was the same as the results of Johansen *et al.* study.^13^

The results of our study showed no correlation between serum leptin (and adjusted leptin) with calcium, phosphorus, parathyroid hormone (PTH) in both genders, but mean leptin in female patients who had serum calcium lower than 8 mg/dl was significantly less than serum leptin of the female patients who had serum calcium level equal or upper than 8 mg/ml. The same result was not obtained in male patients.

Our study showed a moderate positive correlation between serum leptin and urea and creatinine in hemodialysis women. This correlation is not seen in male patients, which was the same as the Johansen *et al.* study.^13^ We did not find any correlation between serum leptin and *KT* /*V* index, which was same as that shown by Koo *et al.*^12^

In Carmona study, a poor correlation was found between increased serum leptin and the markers of malnutrition in hemodialysis patients.^11^

In diabetic hemodialysis patients in this study, no correlation between serum leptin and other variables was found. In nondiabetic patients the correlation was found only between leptin and age. Only 12 diabetic patients were included in our study.

Considering the results of our study and the pervious ones, we noticed varying results. Some of these differences are due to different methods and kits which are used for measurement of leptin. Some studies measured serum leptin and some others measured plasma leptin. Also some measured free serum leptin and sometimes leptin bounded to receptors. Besides the population which were studied differed from each other in their inflammatory status and glucocorticoid usage in last 3 months or taking erythropoietin which may alter serum leptin. Since chronic hemodialysis patient have to take different drugs affecting on iron, calcium, phosphorous, we cannot ignore the effect of these drugs on the laboratory parameters. As shown in Tables 2 and 4, our patients were not severely malnourished.

According to all the conditions mentioned, it can be concluded that serum leptin level cannot be considered as a marker for malnutrition in hemodialysis patients and more studies should be done with larger sample size that include healthy control group.
